# TREADS: tyre nanoparticles produced using a bench-top tyre wear simulator

**DOI:** 10.1039/d6en00217j

**Published:** 2026-07-02

**Authors:** David P. O'Loughlin, Charlotte Gisbourne, Coco Day, Joe Beeby, Tom O'Neill, Molly J. Haugen, Nobuhiro Morone, Evert Duistermaat, Renée de Boeck, Sebastiaan H. Galesloot, Jos van Triel, Miriam Gerlofs-Nijland, Flemming Cassee, Anne E. Willis, Adam M. Boies, Marion MacFarlane

**Affiliations:** a MRC Toxicology Unit Gleeson Building, Tennis Court Road Cambridge CB2 1QR UK mm2312@cam.ac.uk; b Department of Engineering, University of Cambridge Trumpington Street Cambridge CB2 1PZ UK; c Independent Consultant Cambridge UK; d RIVM Dutch National Institute for Public Health and the Environment Bilthoven The Netherlands; e Institute for Risk Assessment Sciences – Division Toxicology, Utrecht University Utrecht The Netherlands

## Abstract

TREADS, the Tyre Rubber Emission And Debris System, is an instrument that can produce high concentrations of sub-micron tyre wear particles, with a size distribution that matches established literature descriptions of nano-tyre particles, in a portable and adaptable device that is compatible with cell exposure systems for toxicological assessment. Test cycles of one-hour with TREADS showed a constant particle concentration averaging between 2–6 × 10^6^ cm^−3^, depending on the tyre sampled. These particles were round, consistent with a gas-to-particle conversion and were produced at rubber temperatures between 37 and 44 °C. TREADS is the first low-cost, bench-top and portable system able to produce nano-TPs and is designed to be reproducible and user-friendly, allowing for hour-long generation of nano-TPs free from any environmental or road surface contamination.

## Abstract 2

TREADS, the Tyre Rubber Emission And Debris System is a bench-top tyre wear particle generator that produces high concentrations of sub-micron particles in an adaptable device that is compatible with cell exposure systems for toxicological assessment.

## Graphical abstract

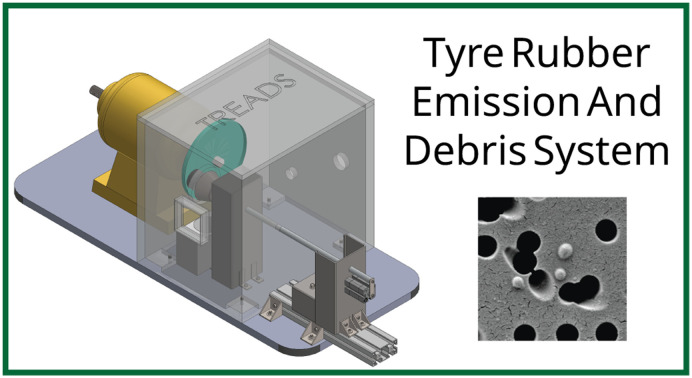

**Environmental significance**

When evaluating the toxicity of tyre wear particles, a large number of studies rely on cryo-milled tyre tread produced by one or two centralised laboratories. These particles may not truly represent the chemistry and morphology of real-world tyre emissions due to their production under cold temperatures, where reactive oxidation chemistry does not occur. This research presents a solution, a low-cost bench-top tyre wear nanoparticle generator that allows for high concentrations of nano-tyre particle aerosols to be generated and sustained for hour long exposures. These particles are generated free from confounding road surface material to allow for comparative toxicological assessment of different tyre formulations and at temperatures matching on-road driving conditions.

## 1 Introduction

Tyre wear particles (TWP) are rapidly becoming an important contributor to air, water and soil pollution and their potential health effects are poorly understood.^1^ In recent work, Zheng *et al.*, 2025 (ref. 2) estimated that 79.5 kt of tyre wear emissions are released in the United Kingdom annually, and of this 4 kt enters our atmosphere. There is increasing interest in studying the potential health effects of TWP due to their ubiquitous presence in the environment and poorly understood impact on our terrestrial and aquatic environments.^3,4^ Despite early evidence indicating that tyre particles have pro-inflammatory and cytokine stimulating effects, the lack of availability of standardised particles and exposure techniques, supported by epidemiology makes drawing any useful population-level conclusions a near impossibility.^1,3^ Tyre wear particles can be broadly classified into three sub-types: road particles (RP) or tyre and road wear particles (TRWP) are particles collected from real-world driving conditions and contain both environmental contaminants adsorbed onto the surface and road wear material. TWP are generated in laboratory conditions on a simulated driving course and may contain road wear depending on the testing surface. These can be generated on a whole tyre dynamometer or other similar device.^5–8^ Tyre or tread particles (TP) are particles made from pure tyre tread, often made by mechanically grinding or cryo-milling tyre samples.^9–11^ In their review, Christou and colleagues summarised the literature pertaining to automotive brake and tyre wear toxicology and found that cryo-milling and rolling roads were used in nearly every *in vitro* tyre toxicology study (48% each) and cryo-milling was used in the majority of *in vivo* experiments.^12^ There were just five studies included in their review that exposed airway epithelial cells to brake or tyre particles, and just one that characterised the sub-micron emissions from tyres.^13^

To date, the apparatus for generating micro- or nano-sized tyre wear particles in the aforementioned studies are large and expensive, often requiring dedicated laboratories and technical staff. Road simulators allow for whole tyres, and real world-like road surfaces to be used, however these instruments are often designed to test tyre characteristics through simulated real-world driving conditions rather than being optimised for particle collection, leading to lower-than-optimal recovery.^14–16^ Cryo-milling is often used for maximum particle mass generation for toxicological studies, with good control of the particle size distribution; however, these particles are generated through a low-temperature event, and their morphology can be very different to those generated on-road, where any oxidative chemistry that takes place during generation may not occur.^11,17,18^

Tyre nanoparticles have been previously measured in both on-road experiments^19,20^ and using road-simulators^8,14,21^ with particle size distributions between 15–110 nm. Tyre-derived nanoparticles are extremely low mass, and were not included in the mass release calculations by Zheng *et al.*, 2025.^2^ These nano tyre particles are likely formed through a gas-to-particle conversion of evaporated tyre components such as extender oils, polymer monomers or hydrocarbons in the tyre tread through a temperature-dependent process.^14,19,22^

One of the primary challenges in TWP research is the lack of an accessible, reproducible method to generate TWP for inhalation toxicological studies, especially in the sub-micron size distribution.^23^ TREADS – the Tyre Rubber Emission And Debris System has been designed to generate and collect sub-micron tyre/tread particles (TP) free from environmental or road surface contamination, but generated through a laboratory friction wear instrument similar to TWP. The instrument is designed to create particles with a size distribution that matches existing literature reports for sub-micron TP, but in a bench-top instrument which could be portable and adaptable to work with existing particle analysis or exposure systems for *in vitro* toxicological assessment. TREADS is accessible, runs on a bench-top and does not require extensive training or specialist equipment. Primarily constructed from off-the-shelf components, TREADS aims to reduce the barrier to TP/TWP research by providing a comparatively low-cost technical solution. In this research, we show that it is possible to use a bench-top device to generate stable, representative nano-TP samples from a range of tyres over a prolonged testing period without the requirement for large, expensive equipment.

## 2 Methods and materials

### 2.1 Tyres tested

To investigate the particle size distribution of nano-tyre particles (nano-TP) from different tyre types, three tyres were selected:

• Michelin Primacy 4+ (205/55R16 91V)

• Yokohama Blu Earth-4s (225/60R17 103V)

• Taurus High Performance (205/55R16 91H)

All samples were taken from new, undriven tyres with no running-in or conditioning. These tyres represented a premium, mid-range and budget option respectively. All tyres were compatible with a standard sports utility vehicle and were available on the market in the UK. All three tyres tested were rated “C” for Rolling Resistance according to the Standard Tyre Label required by Regulation (EU) 2020/740. For Wet Gripping, the Michelin was rated “A”, the Yokohama “B” and the Taurus “C”. The tyres were sampled for analysis by cutting a 35 mm diameter “puck” through the full thickness of the tyre. The pucks were rinsed with deionised water to remove any rubber flakes or cutting debris and mounted to a 70 mm diameter acrylic disc by gluing the tyre to the acrylic disc, tread surface out. This could then be loaded into TREADS and secured with a machine screw.

### 2.2 Tyre particle generator – TREADS

**Fig. 1:**
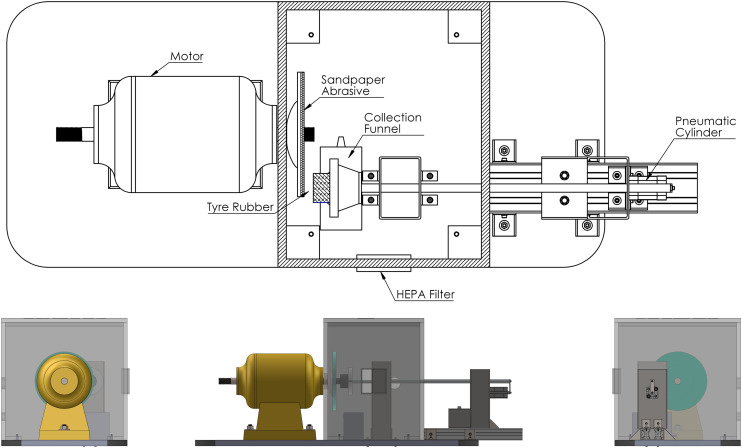
Labelled top-view schematic and technical rendering of TREADS: Tyre Rubber Emission and Debris System.

TREADS is a newly designed tyre wear simulator which uses samples of tyres rather than a whole tyre sample, in contrast to previous designs, and is shown as Fig. 1.^5,16,24^ TREADS generates particles by spinning a sandpaper abrasive against a static rubber puck, which is pressed against it by a pneumatic cylinder. TREADS utilises a single speed motor running at 2850 rpm which spins a silicon carbide sandpaper abrasive (RS PRO 188-3420 P400 grit, with an approximate abrasive size of 35.0 ± 1.5 μm) mounted to a PVC disc for its better impact and wear resistance compared to the acrylic enclosure. Silicon carbide (SiC) was selected over aluminium oxide (Al_2_O_3_) as SiC nanoparticles have been shown to have low to no cytotoxicity in both *in vitro* and *in vivo* studies, although this is dependent on particle size, morphology and dose.^25,26^ The effective linear speed of the tyre is 8.65 m s^−1^ at the furthest edge of the rubber from the centre of the rotation and 19.1 m s^−1^ at the nearest edge, which is between 31.14 and 68.76 km h^−1^. The collection enclosure has a volume of 22.5 L. Particles are collected through a 3D-printed funnel block which could interface with tubing. The funnel position was optimised using COMSOL physics modelling software using the laminar flow and particle tracking for fluid flow simulation modules to find the optimum position for the collection funnel, based on the limitations of the geometry of the enclosing box and support structures contained within. The funnel was printed from SLS Nylon PA12, with a vibro-polished finish to reduce surface roughness.

Similar to the limitations highlighted by Kim *et al.*, 2018,^5^ TREADS was not designed to mimic real-world driving conditions precisely but to maximise the amount of wear material produced so that it could be used in toxicological or environmental research. Thus, the system most closely simulated an anti-lock braking event, where a tyre would slip against the road surface repeatedly. It was considered more important to regulate the rubber temperature to ensure that particles were generated in a manner more closely representing real-world driving, especially cornering, braking and accelerating. The particle generation procedure involved spinning the motor up to full speed and taking a number of blank measurements. TP generation occurred by pulsing the tyre material onto the abrasive using a pneumatic cylinder and computer-controlled solenoids. This was done to prevent the tyre rubber overheating by maintaining realistic core rubber temperatures measured by an internal thermocouple.

For particle generation tests, sampling took place over one hour to investigate whether it was possible to design long exposure runs for future integration with toxicology exposure systems. A pneumatic cylinder applied load to the rubber surface and was set to 0.38 bar gauge, which applies a force of 7.45 N. Across the 35 mm tyre puck, this is equivalent to 8 kPa. The cylinder was pulsed, with 0.5 seconds of contact time followed by 1 second of rest. This was repeated 10 times, followed by 45 seconds of rest to control rubber temperature. This minute long process was repeated for a total duration of 10 minutes. At the end of the 10 minute window, the pulses were stopped but the instruments remained sampling until the particle number concentration within the enclosure returned to baseline levels. At this point, the sandpaper was refreshed. This cycle was repeated four times, resulting in a total duration of one-hour.

### 2.3 Instruments

**Fig. 2:**
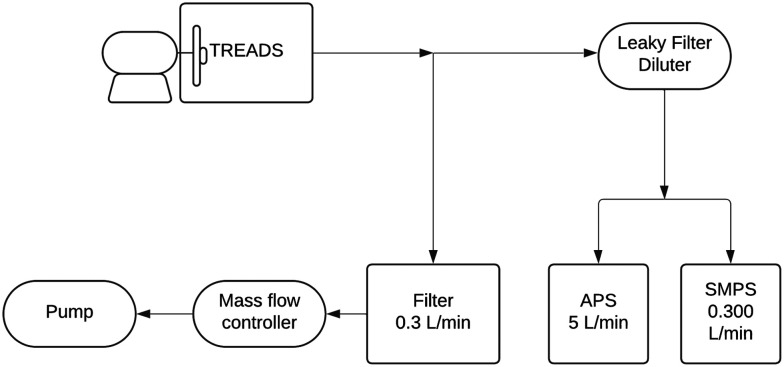
Particle generation methodology using TREADS, SMPS and APS.

A scanning mobility particle sizer (SMPS), consisting of an electrostatic classifier (TSI Inc., 3082) with a dynamic mobility analyser (DMA) (TSI Inc., 3081) and a high concentration condensation particle counter (CPC) (TSI Inc., 3752), was used to measure particle sizes between 17.2 to 542.5 nm with a 50 s scan time. Sheath and sample flows were maintained at 3.0 L min^−1^ and 0.265 L min^−1^, respectively. A soft X-ray neutraliser (TSI Inc., 3088) was used to establish charge equilibrium (Fig. 2).

Particle size distributions between 0.5 μm and 19.8 μm were measured with an aerodynamic particle sizer (APS) (TSI Inc., 3321). The APS operated a total flow of 5.0 L min^−1^ and a sheath flow of 4.0 L min^−1^. All particle results are expressed as the normalised particle number concentration, d*N*/dlog *D*_p_.

### 2.4 Scanning electron microscopy

For Scanning Electron Microscopy (SEM), particles were collected on 37 mm polycarbonate membrane filters with a pore size of 0.4 μm (SKC, 225-1609), and PTFE filters with a pore size of 0.2 μm (Whatman, WHA7582004) and analysed by SEM (FEI Quanta 250FEG SEM, Thermo Fischer Scientific, Oregon USA). Filter papers were sputter coated in-house with gold (EMITECH k950 K, Quorum Technologies, Kent, UK), following a standard procedure (1 × 10^−1^ mbar of Argon gas, 20 mA, for 2 min, coating thickness approximately 10 nm). ImageJ was used to analyse particle size from SEM images.

### 2.5 Hardness and rheology

Shore A hardness was tested using a Sauter HBA Analogue Shore Hardness Tester with test stand, following the manufacturers instructions. Measurements were taken at three points on the tread surface of each sample. Rheology experiments were performed on an Anton-Paar MCR302 Rheometer at the Thermal Characterisation Facility, University of Cambridge. A frequency sweep was conducted with a fixed strain of 0.1% between 0.1 and 100 rad s^−1^ at 25 °C. The rheometer recorded storage modulus (*G*′), loss modulus (*G*″) and complex viscosity (η*) and tan *δ* was calculated.

### 2.6 Leaky filter diluter

To protect particle instruments from overload due to high concentrations, a leaky filter diluter (LFD) was constructed, following Collins (2010).^27^ This was necessary as initial experiments with an AlphaSense OPC showed that after 100 seconds, the sensor would saturate, returning a 0 value. The diluter was validated using a silver particle generator (SPG),^28^ and a differential mobility analyser (DMA) was used to select particle size ranges. The schematic and dilution factor of the LFD are shown in SI (Fig. S1). The LFD had a volumetric dilution factor of 7 on average and was stable between 10 and 150 nm.

## 3 Results

### 3.1 Background particle number

Background measurements were taken on the SMPS prior to every TWP generation experiment. The motor was allowed to spin up to full-speed, and three measurements were taken to verify the baseline for experimental runs. Under these conditions, particle concentrations across the measured size range were stable and low, with the highest concentration around 400 cm^−3^, below indoor background concentrations, which range from 1500–1600 cm^−3^.^29^ The measured background concentrations are plotted in SI (Fig. S2). The background level was four orders of magnitude below the undiluted concentrations of TP produced by TREADS.

### 3.2 Tyre particle generation

**Fig. 3:**
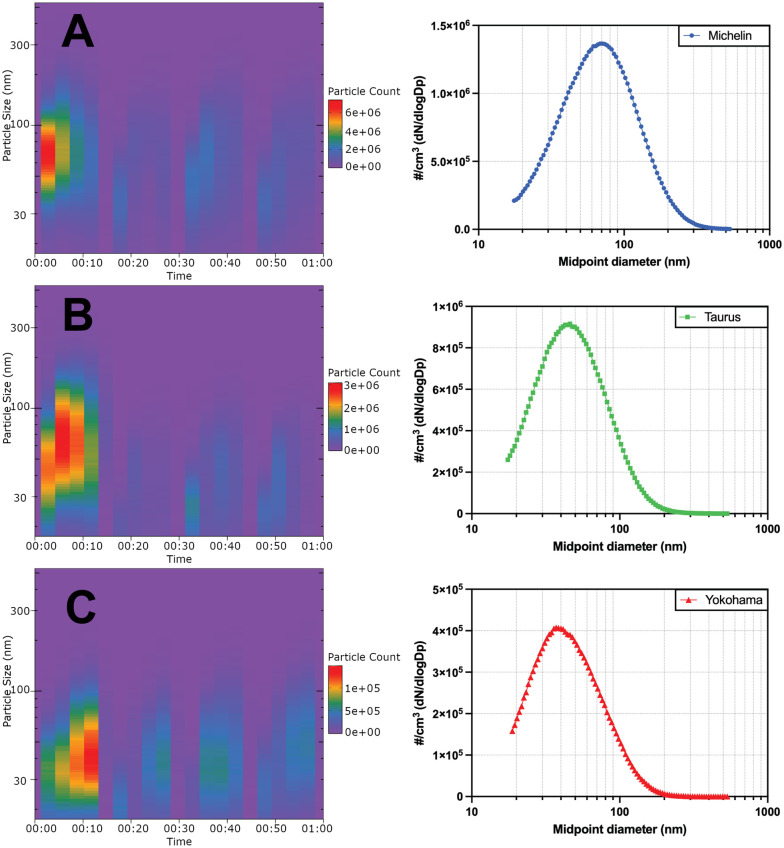
Representative particle size information per test run for A – Michelin, B – Taurus and C – Yokohama tyre samples. Left: Data shown are time-resolved particle size graphs with time on the *X*-axis, size on the *Y*-axis and concentration on the *Z*-axis. Right: Normalised particle number size concentration with particle size on the *X*-axis and concentration on the *Y*-axis. All data shown are LFD diluted to protect instruments.

Representative particle number size distributions from SMPS measurements for the three tyres tested using TREADS are shown in Fig. 3. Using TREADS, three tyres from 3 manufacturers (budget, midrange, and premium) were tested using the same testing regime to assess differences in nano-TP evolution. TREADS is size-selective, producing high concentrations of nano-TPs, while almost no particles above 500 nm were detected (APS measurements in SI, Fig. S3). Larger, mechanically abraded rubber fragments were also produced by TREADS and were observed visually as rubber chunks 2–5 mm in length. These larger fragments were thrown around the enclosure but were not airborne and did not enter the collection funnel.

Average undiluted particle number concentrations for the Michelin, Taurus and Yokohama tyres were 6.37 × 10^6^ cm^−3^, 3.80 × 10^6^ cm^−3^ and 2.75 × 10^6^ cm^−3^ respectively over the 1 hour testing cycle as summarised in Table 1. The density was assumed to be 1.2 g cm^−3^ as the particles are believed to be predominantly derived from the volatile components of tyre rubber.^14,30^

**Table 1:** Summary of particle concentrations and rubber internal temperature produced by TREADS

	Diluted (measured) number concentration (×10^5^ cm^−3^)	Undiluted (calculated) number concentration (×10^6^ cm^−3^)	Diluted (measured) mass concentration (μg m^−3^)	Undiluted (calculated) mass concentration (μg m^−3^)	Average internal temperature
Michelin	8.24	6.37	500	3864	43.8 °C
Taurus	5.05	3.80	168	1296	37.8 °C
Yokohama	2.54	2.75	53	412	39.7 °C

Undiluted concentrations were estimated by multiplying the measured concentration by the constant dilution factor (7.7) calculated for the LFD.

**Fig. 4:**
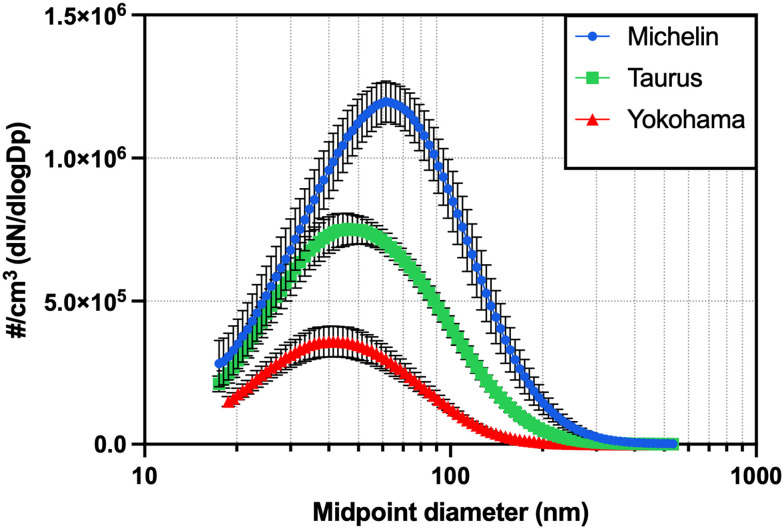
Average (*n* = 4 per tyre) normalised particle number size concentration from Michelin Taurus and Yokohama tyres with standard deviation. Particle size on the *X*-axis and concentration on the *Y*-axis. All data shown are LFD diluted to protect instruments.

In all experiments, a higher concentration of tyre wear particle emissions was observed during the first testing cycle. As each of the four cycles used refreshed sandpaper, this was attributed to the tyre or some coating thereof, rather than the abrasive. This initial emission may be caused by a mould release agent or other protective coating used during manufacture.^31^ For the Michelin and Taurus tyres, a shift in the PSD was observed from ≈70 nm for the first sandpaper discs to ≈40 nm when particle size distribution (PSD) stabilised. Fig. 4 shows normalised particle number size distributions for the three tyres tested (*n* = 4) over the one-hour test cycle. The mobility diameter of these particles is consistent with previous works from Mathissen *et al.*, 2011 (ref. 19) and Kwak *et al.*, 2014,^20^ who studied the evolution of nano-TPs from real-world driving conditions including cornering, braking and harsh accelerating. The main advantage of the TREADS system is that the TWP concentration produced was much higher. The Michelin samples peaked at an average measured normalised particle concentration of 1.2 × 10^6^ cm^−3^ (diluted) equating to a calculated undiluted peak concentration of 9.3 × 10^6^ cm^−3^; conversely, the highest peak reported by Zhong *et al.*, 2024 (ref. 32) using a road simulator was around 1.0 × 10^6^ cm^−3^, which may also have contained particles associated with the road surface.^32^ A comparison of particles produced by TREADS to other published techniques is shown in Table 2. The particle diameters are summarised in Table 3; the distributions were approximately log-normal, and slightly right-skewed. As shown in Fig. 4, the size distributions for nano-TPs generated by TREADS were unimodal over the measured particle-size range. The modal particle sizes were 61.5 nm for the Michelin tyre, 46.1 nm for the Taurus tyre, and 42.9 nm for the Yokohama tyre. The particles generated by TREADS may initially form through homogeneous nucleation and subsequently grow by gas–particle partitioning and/or coagulation before measurement, resulting in a single accumulation of particles within the measured size-range. The Michelin tyre mode particle number size-matched Kwak *et al.*, 2014,^20^ while the Yokohama and Taurus were slightly smaller. During the hour-long test cycle, the average undiluted mass concentration for the Michelin tyre was 3864 μg m^−3^, 1296 μg m^−3^ for the Taurus, and 412 μg m^−3^ for the Yokohama. Calculated mass flow rates for the Michelin, Taurus and Yokohama tyres were 0.34 μg s^−1^, 0.12 μg s^−1^, and 0.037 μg s^−1^, respectively. The PSD of all three tyres tested is predominantly in the Aitken mode, which agrees with the suggested generation mechanism of evaporation of volatile tyre components, followed by a gas-to-particle conversion.^21,34^

**Table 2:** Comparison of particles produced by TREADS to other literature nano-TPs, adapted from Zhong *et al.*, 2024 (ref. 32)

References	Method	Instruments	Size distribution
TREADS	Pin-on-disc	SMPS + APS	Unimodal 17–200 nm
Zhong *et al.*, 2024 (ref. 32)	Road simulator (drum)	ELPI+	Bimodal, 10–13 nm and 23–41 nm
Dall'Osto *et al.*, 2014 (ref. 33)	Road simulator (drum)	SMPS + APS	Bimodal, peaks at 35 nm and 85 nm
Mathissen *et al.*, 2011 (ref. 19)	On road	EEPS	Speed dependent: low speed unimodal at 70–90 nm, high speed biomodal at <10 nm and 30–60 nm
Kreider *et al.*, 2010 (ref. 9)	Road cleaning	TOM	Unimodal 50–75 μm
Dahl *et al.*, 2006 (ref. 14)	Road simulator (track)	SMPS + CPC	Unimodal, condition dependent, means between 15–50 nm

**Table 3:** Summary of particle diameters produced by TREADS

	Mode particle diameter (nm)	Count median diameter (nm)	Mass median diameter (nm)	Diameter of average mass (nm)
Michelin	61.50	59.07	157.10	96.33
Taurus	46.10	51.51	129.80	81.77
Yokohama	42.90	40.57	85.73	58.97

The differences between concentrations of nano-TPs may also have been influenced by differences in tread pattern between the tyres sampled. Each tyre was sampled from the same tread section to ensure that the tread pattern was the same between pucks sampled from the same tyre. The Michelin tyre was characterised by four circumferential grooves with diagonal stripes across the surface. The Yokohama tyre has a dense V-shaped pattern with many narrow grooves, while the Taurus tyre had wide circumferential grooves and larger tread blocks with fewer sipes. These differences in tread pattern will affect the contact area between the tyre and the abrasive and may play a role in the concentration of nano-TPs released.

The nano-TPs generated by TREADS should not be interpreted solely as mechanical abrasion of tyre tread into solid nanoparticles as has been observed in other studies. The particles observed by Kwak *et al.*, 2014 (ref. 20) had a morphology more consistent with particles generated through physical abrasion. However on-road experiments have shown that nanoparticles can form through evaporation of volatile or semi-volatile components of tyre rubber material. This occurs during harsh braking or cornering, by volatilisation of extender oils or polymer monomers, followed by subsequent nucleation or condensation.^20^

This distinction between solid and volatile particles is important from a toxicological perspective as freshly formed volatile or semi-volatile particles may differ from solid TWP in their chemical composition, volatility, surface chemistry, oxidative potential and persistence, in the context of inhalation exposure.^20,21,35^

### 3.3 Rubber temperature

**Fig. 5:**
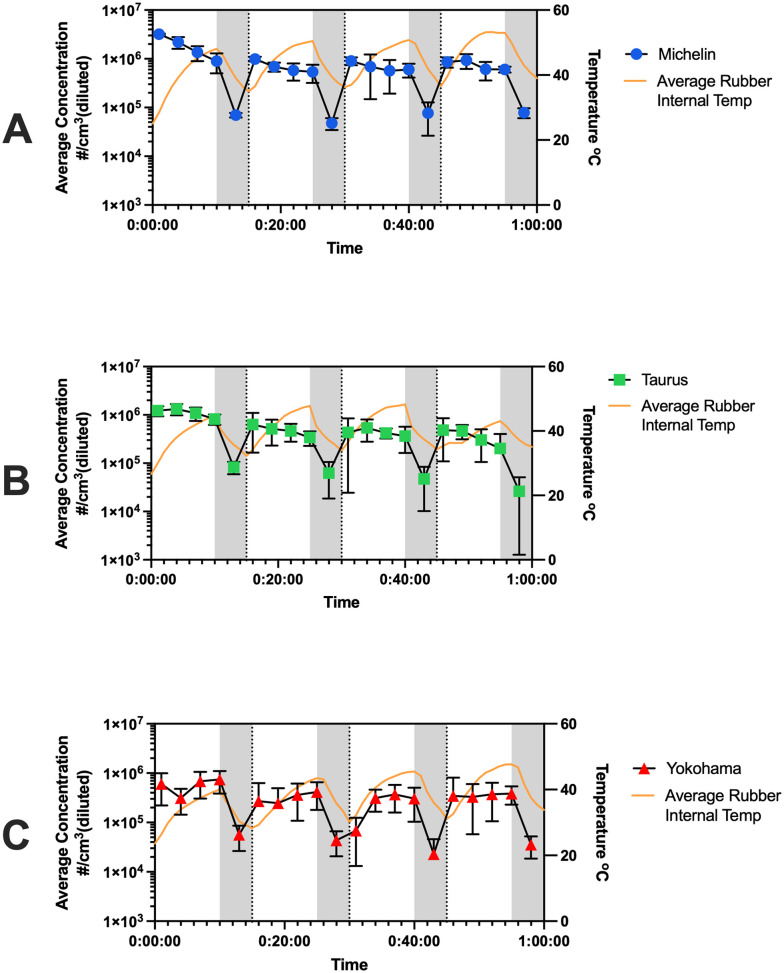
Average (*n* = 4 per tyre) normalised particle number concentration from Michelin (A) Taurus (B) and Yokohama (C) tyres with average rubber internal temperature. Data are shown as time-resolved particle number concentration graphs, with experiment time on the *X*-axis and average number concentration with standard deviation on the *Y*-axis. All data shown are LFD diluted to protect instruments. Grey shaded regions indicate experiment time without motor running, with no particle generation, to allow particle number concentration to return to baseline. Sandpaper was refreshed at each dotted line.

**Fig. 6:**
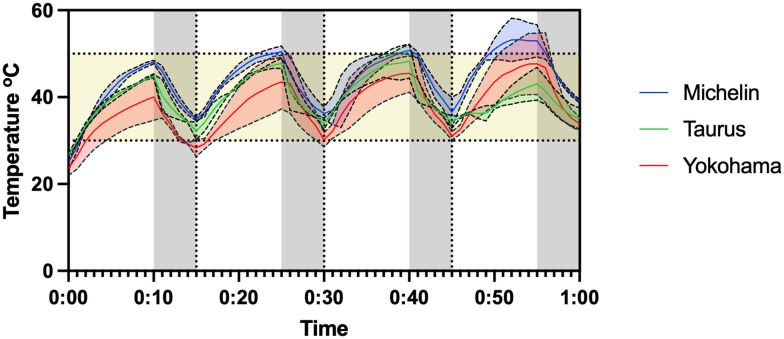
Average temperature with standard deviation for the three test tyres. The *X*-axis shows time in minutes with the testing cycle highlighted, and the *Y*-axis shows temperature in degrees celsius with the standard driving temperature highlighted by the yellow box. Grey shaded regions indicate experiment time without motor running, with no particle generation, to allow particle number concentration to return to baseline. Sandpaper was refreshed at each dotted line.

While TREADS was not designed to mimic road conditions, maintaining a realistic tyre temperature was vital due to the relationship between temperature and particle emission, with higher temperatures increasing the particle emission factor.^6,36^ Additionally, TWP may undergo structural and chemical changes at extremes of temperature.^3,9,37^ The rubber samples were instrumented with an internal thermocouple, which allowed for the rubber internal temperature to be monitored during particle generation. This is to ensure runs are designed to simulate real-world driving conditions without needing extremes of temperature to produce nano-TPs, as used by Park *et al.*, 2017.^38^ The average particle number concentration during experiments with average rubber internal temperature is shown as Fig. 5. Fig. 6 shows the average internal rubber temperature through the hour-long tests, with a highlighted band showing an expected tyre temperature range. Temperatures of 30–50 °C were reported by Chang *et al.*, 2020 (ref. 6) during tyre dynamometer experiments, while the highest on-road temperature with similar tyre sizes was 52 °C.^39^

Average internal rubber temperature was measured using a thermocouple placed 2–3 mm below the tread surface, as close as was practical without damaging the thermocouple. For the Michelin tyre the average internal temperature was 43.8 °C, for the Taurus 37.8 °C, and 39.7 °C for the Yokohama.

As discussed earlier, the primary generation mechanism for particles from TREADS is likely evaporation followed by condensation or nucleation of evaporated extender oils, rubber monomers or hydrocarbons in the tyre tread through a temperature-dependent process.^14,19,22^ Rubber is a viscoelastic material with poor thermal conductivity which undergoes repeated cycles of deformation and recovery during abrasion where mechanical energy is converted to heat by viscoelastic hysteresis. Local hotspots can form at the contact point between the tyre rubber and abrasive and this localised heat flux is likely more important to the formation of nano-TPs than overall rubber temperature.^40–42^ The rubber “blackbody” temperatures reported here are used purely as an indicator that the abrasive conditions during TREADS operation are not superheating the rubber to the point of evaporation as has been reported in other work.^38^ The generation of nano-TPs, therefore, is likely driven by a combination of rubber surface temperature and tyre rubber formulation. The Taurus tyre showed a higher total particle number concentration than the Yokohama at a lower temperature, which could be explained by a greater abundance of volatile or semi-volatile components in the rubber, such as extender oils, waxes or degradation products. While this observation is consistent with the experimental data, it cannot be confirmed without direct chemical characterisation of the tyre, or identity of ingredients used in manufacture. This information is proprietary, and may contain hundreds to thousands of different chemicals, either added directly or as contaminants in feedstocks.^43^

### 3.4 Morphological analysis of nano-TPs

**Fig. 7:**
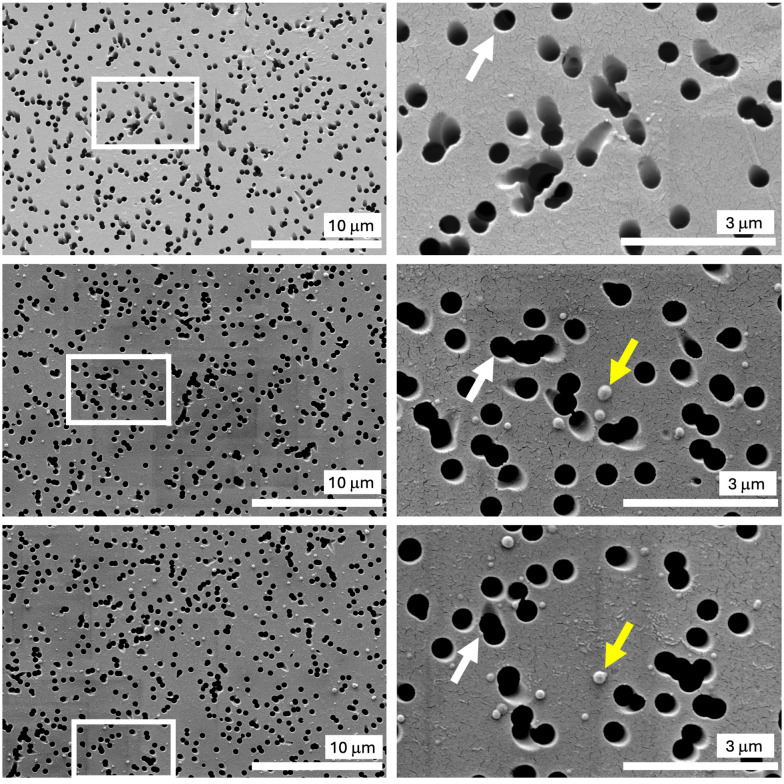
SEM micrographs of tyre wear nanoparticles generated with TREADS using the Michelin tyre. Particles were collected onto 37 mm polycarbonate filters with 0.4 μm pore size (SKC Ltd, UK). Top: Blank polycarbonate filter, middle and bottom: images from the Michelin tyre. White up arrows indicate pores in filter membrane, while yellow down arrows indicate tyre particles.

Polycarbonate filter membranes were exposed to particles generated by TREADS for the one-hour test cycles where any flow not used for instrumental analysis was diverted onto the filter. Membranes were gold sputter coated and representative images of tyre nanoparticles are shown in Fig. 7. Blank control filters were collected by imaging clean air control filters. SEM micrographs of nano-TP revealed particles with a size distribution, with no particles larger than about 300 nm being observed. This matched the size distribution measured by the SMPS. The particles exhibit a round, uniform morphology consistent with a particle generated through a gas-to-particle conversion following an evaporative mechanism through condensation/coagulation of volatile, or semi-volatile organic components of tyre tread.^21,34^

This morphology agrees with nanoparticles produced by Kim *et al.*, 2018,^5^ where smaller particles have a more round morphology while micron-sized particles, produced through an abrasive process, tend to be more jagged and elongated.^5^ However, the surface of the filter is much rougher and fibrous, and it was not possible to distinguish any deposited tyre particles from the background. In both experiments, the filters were also weighed before and after exposure using an analytical balance and no change in mass greater than the percentage measurement error was observed.

### 3.5 Comparison of shore hardness and temperature with particle number concentration

**Fig. 8:**
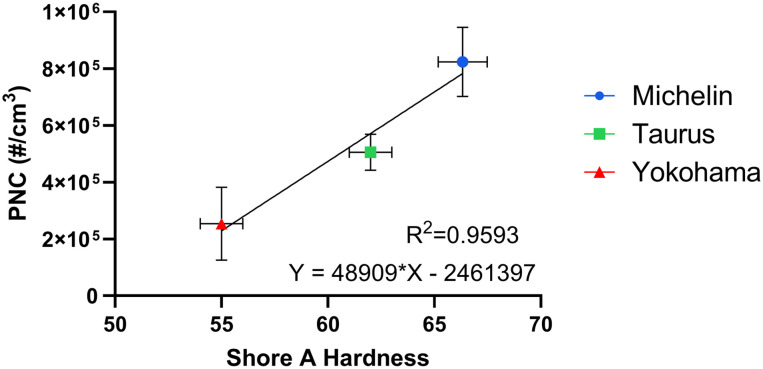
Average particle number concentration plotted against shore A hardness (top) and temperature (bottom). *X*-Axis shows test parameter (hardness in shore A units) and *Y*-axis shows average particle number concentration in cm^−3^.

During operation, it was observed that while the Michelin tyre produced the highest concentration of nanoparticles, it also deposited the lowest amount of macro-rubber material in the TREADS enclosure. To understand this effect more, rubber hardness was measured on the Shore A scale, and linear regression used to test whether rubber hardness, impacted the average particle number concentration. This gave a strong correlation of 0.96; however, more tyres would need to be tested (increasing *N*) in order to draw a statistical conclusion. This may explain the difference in overall particle number concentration and may have an important impact in future regulatory discussions around tyre wear emissions. The relationship between rubber hardness and average particle number concentration is shown as Fig. 8.

**Fig. 9:**
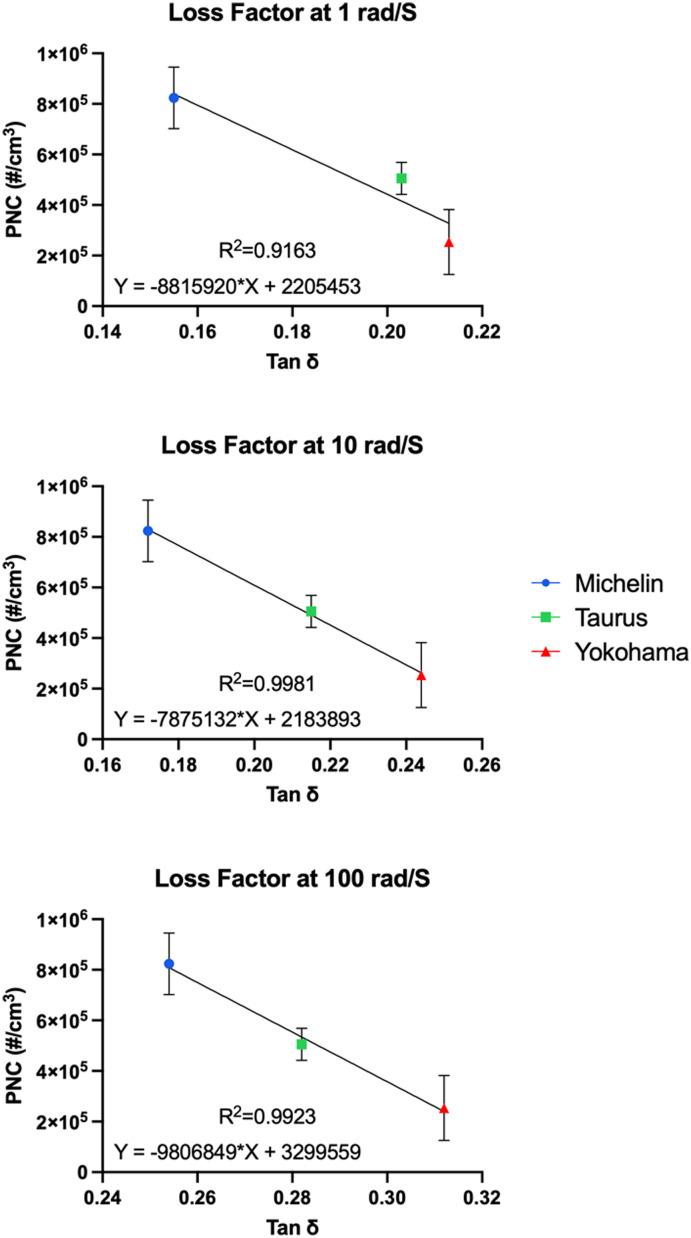
tan *δ* for Michelin, Taurus and Yokohama test tyres plotted against particle number concentration at different angular frequencies. *X*-Axis is angular frequency in rad s^−1^ and *Y*-axis is tan *δ*.

Subsequently, it was decided to measure the tan *δ* of the rubber samples using an Anton Parr Rheometer. tan *δ* is a metric that describes the energy loss in viscoelastic materials. To calculate tan *δ*, a frequency sweep was performed at constant temperature and strain. tan *δ* for each of the three test tyres were plotted against particle number concentration at 1, 10, and 100 rad s^−1^. Here too, a strong linear relationship was observed but again more tyres are required to make a definitive conclusion (Fig. 9). As tan *δ* decreases, this suggests that there is a higher concentration of reinforcing filler content which increases elasticity and reduces energy dissipation, decreasing the total particle number concentration.^44^ With current regulations focussing on particulate mass loss (Euro7), this finding may reveal that nano-particle emissions are tied to other tyre properties and are thus a crucial part of tyre emissions currently being overlooked by regulatory and toxicology approaches. Further work is required to characterise the toxicity of these emissions.

## Conclusions

The Tyre Rubber Emission and Debris System (TREADS) is a bench-top tyre particle simulator capable of generating stable, log-normal, nano-tyre particle aerosols for extended sampling periods, allowing for the device to be integrated with toxicological exposure systems. In this work, we have demonstrated the proof of concept and characterised the particle aerosols produced. Further work is required to chemically and elementally characterise these particles and to employ TREADS to assess their inhalation toxicology – these experiments are ongoing.

## Author contributions

David P. O'Loughlin: methodology, investigation, formal analysis, writing – original draft. Charlotte Gisbourne: methodology, resources. Coco Day: methodology, resources. Joe Beeby: methodology, resources. Tom O'Neill: methodology, resources. Molly J. Haugen: resources. Nobuhiro Morone: investigation. Evert Duistermaat: methodology, investigation. Renée de Boeck: methodology, investigation. Sebastiaan H. Galesloot: investigation. Jos van Triel: supervision. Miriam Gerlofs-Nijland: supervision. Flemming Cassee: supervision. Anne E. Willis: supervision, funding acquisition. Adam M. Boies: supervision, funding acquisition, writing – review & editing. Marion MacFarlane: supervision, project administration, funding acquisition, writing – review & editing.

## Conflicts of interest

There are no conflicts to declare.

## Supplementary Material

EN-013-D6EN00217J-s001

## Data Availability

Data for this article are available *via* Open Science Framework at https://doi.org/10.17605/OSF.IO/Z6WJ2. Supplementary information (SI) is available. See DOI: https://doi.org/10.1039/d6en00217j.
