# Supplementary material for: TREADS: tyre nanoparticles produced using a bench-top tyre wear simulator
Source: Environ Sci Nano. 2026 Jul 2;13(7):3108–18. doi: 10.1039/d6en00217j (PMC13325192; doi:10.1039/d6en00217j)
Supplement: EN-013-D6EN00217J-s001 [file EN-013-D6EN00217J-s001.pdf]

Supplementary Information (SI) for Environmental Science: Nano.
This journal is © The Royal Society of Chemistry 2026

TREADS: Tyre Nanoparticles Produced Using a Bench-Top Tyre Wear Simulator

David P. O'Loughlin,*,†,‡ Charlotte Gisbourne,¶ Coco Day,¶ Joe Beeby,¶ Tom O'Neill,¶ Molly J. Haugen,‡ Nobuhiro Morone,† Evert Duistermaat,§ Reneé de Boeck,§ Sebastiaan H. Galesloot,§ Jos Van Triel,§ Miriam Gerlofs-Nijland,§ Flemming Cassee,§ Anne E. Willis,† Adam M. Boies,‡ and Marion MacFarlane*,†

†MRC Toxicology Unit, Gleeson Building, Tennis Court Road, Cambridge, CB2 1QR, United Kingdom

‡Department of Engineering, University of Cambridge, Trumpington Street, Cambridge CB2 1PZ, United Kingdom

¶Independent Consultant, Cambridge, UK

§RIVM Dutch National Institute for Public Health and the Environment, Bilthoven, The Netherlands

E-mail: do362@cam.ac.uk; mm2313@cam.ac.uk

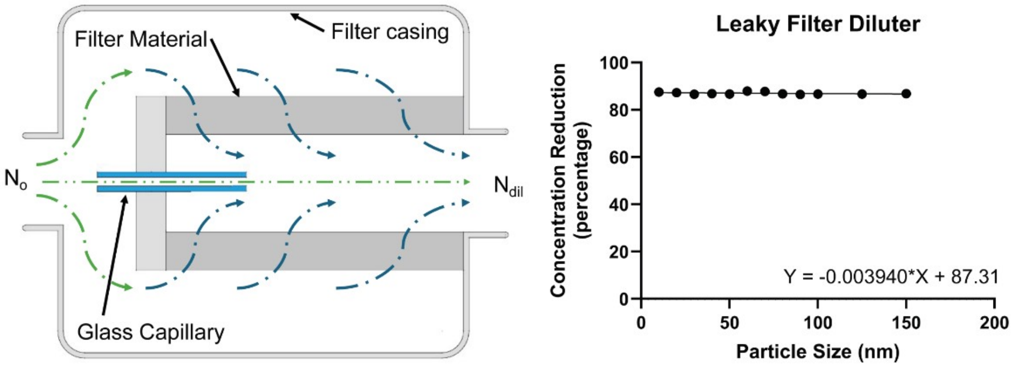

Supplemental 1. Schematic of "Leaky Filter" diluter and percentage concentration reduction between 10 and 150 nm

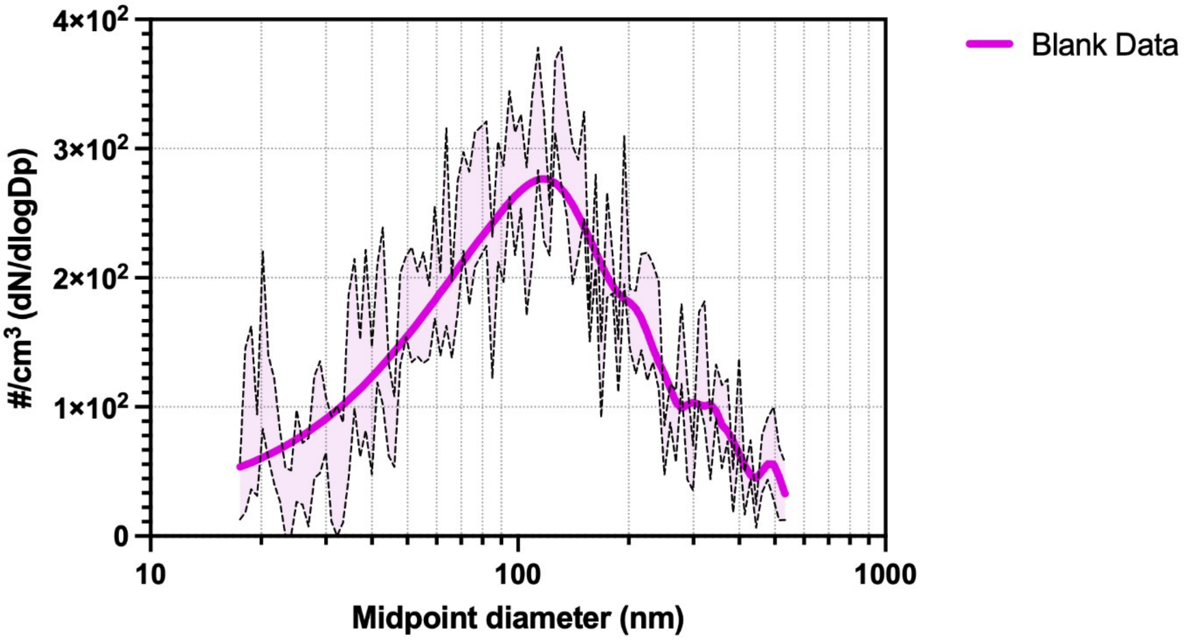

Supplemental 2. Normalised undiluted background SMPS particle number data. LOWESS Smoothed curve with SEM. X-axis shows particle size (diameter midpoint), and Y-axis shows average (n=6, 3 measurements per experiment) normalised particle number concentration, dN/dlogDp. Measurements taken for 6 minutes prior to exposure experiments

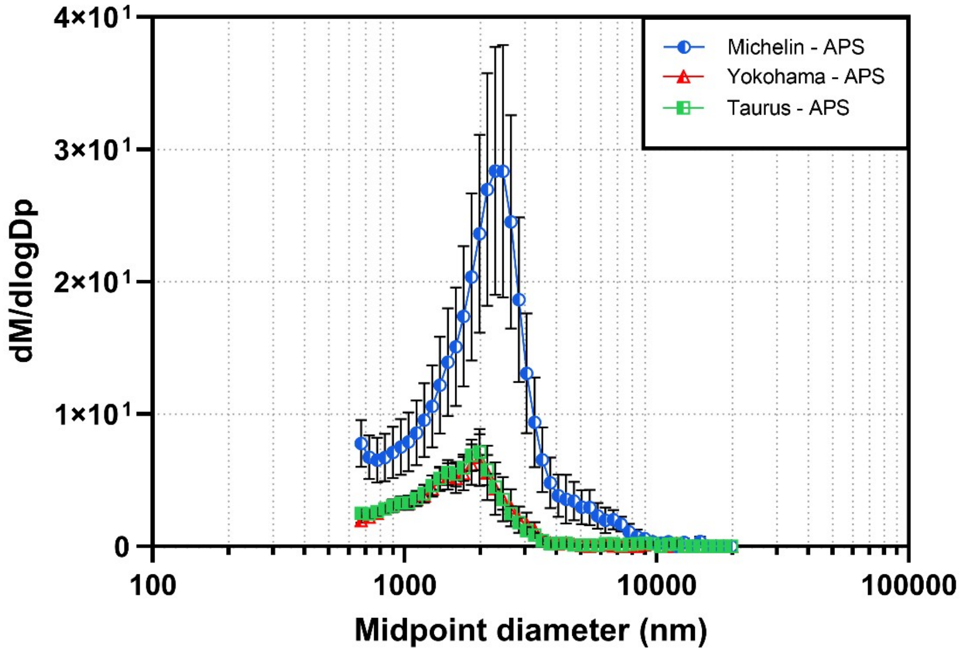

Supplemental 3. Average (n=4 per tyre) normalised particle mass size concentration from Michelin Taurus and Yokohama tyres with standard deviation. Particle size on the x-axis and mass concentration in µg/M³ on the y-axis. All data shown are LFD diluted to protect instruments.

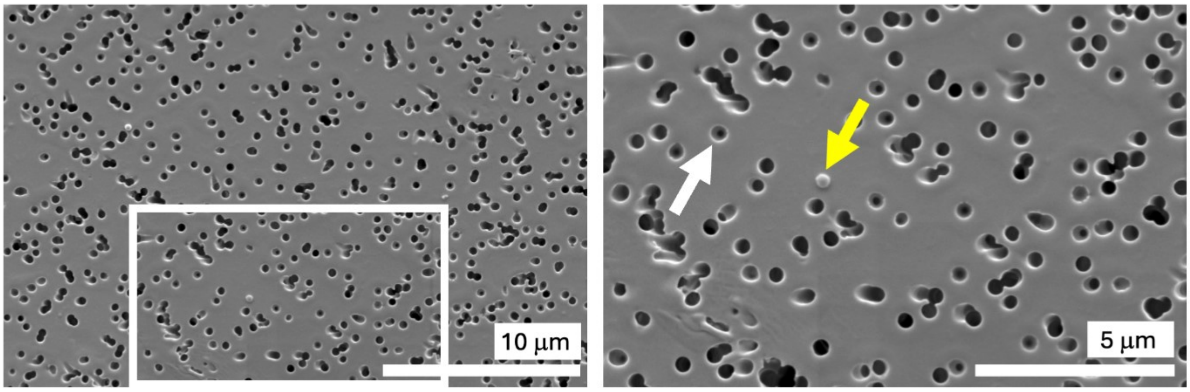

Supplemental 4. EM micrographs of tyre wear nanoparticles generated with TREADS using the Taurus tyre. Particles were collected onto 37 mm Polycarbonate filters with 0.4 µm pore size (SKC Ltd, UK). White up arrows indicate pores in filter membrane, while yellow down arrows indicate tyre particles.
